# Transmission Electron Microscopy Reveals Distinct Macrophage- and Tick Cell-Specific Morphological Stages of *Ehrlichia chaffeensis*

**DOI:** 10.1371/journal.pone.0036749

**Published:** 2012-05-15

**Authors:** Sarah E. Dedonder, Chuanmin Cheng, Lloyd H. Willard, Daniel L. Boyle, Roman R. Ganta

**Affiliations:** 1 Department of Diagnostic Medicine/Pathobiology, College of Veterinary Medicine, Kansas State University, Manhattan, Kansas, United States of America; 2 Division of Biology, College Of Arts And Sciences, Kansas State University, Manhattan, Kansas, United States of America; University of Minnesota, United States of America

## Abstract

**Background:**

*Ehrlichia chaffeensis* is an emerging tick-borne rickettsial pathogen responsible for human monocytic ehrlichiosis. Despite the induction of an active host immune response, the pathogen has evolved to persist in its vertebrate and tick hosts. Understanding how the organism progresses in tick and vertebrate host cells is critical in identifying effective strategies to block the pathogen transmission. Our recent molecular and proteomic studies revealed differences in numerous expressed proteins of the organism during its growth in different host environments.

**Methodology/Principal Findings:**

Transmission electron microscopy analysis was performed to assess morphological changes in the bacterium within macrophages and tick cells. The stages of pathogen progression observed included the attachment of the organism to the host cells, its engulfment and replication within a morulae by binary fission and release of the organisms from infected host cells by complete host cell lysis or by exocytosis. *E. chaffeensis* grown in tick cells was highly pleomorphic and appears to replicate by both binary fission and filamentous type cell divisions. The presence of *Ehrlichia*-like inclusions was also observed within the nucleus of both macrophages and tick cells. This observation was confirmed by confocal microscopy and immunoblot analysis.

**Conclusions/Significance:**

Morphological differences in the pathogen’s progression, replication, and processing within macrophages and tick cells provide further evidence that *E. chaffeensis* employs unique host-cell specific strategies in support of adaptation to vertebrate and tick cell environments.

## Introduction

*Ehrlichia chaffeensis* is a Gram negative obligate intracellular pathogen that is transmitted via the bite of an infected *Amblyomma americanum* tick to humans and several other vertebrate hosts [1]–[3]. This organism is responsible for an emerging disease, human monocytic ehrlichiosis (HME) [4], [5]. HME is characterized by an acute onset of febrile illness which can sometimes be a fatal disease. Clinical symptoms of the disease may resemble flu-like illness which may include malaise, headache, myalgia and persistent fever. Laboratory findings may include leukopenia, thrombocytopenia, and elevated liver transaminases [4]–[6].

*E. chaffeensis* and other related tick transmitted rickettsial pathogens are capable of persisting in both vertebrate and tick hosts [7]–[13]. The pathogens may have evolved unique strategies to establish infections in both invertebrate and vertebrate hosts in order to successfully complete their lifecycle in dual hosts. Persistent infection in ticks is also important as the organism cannot be transovarially transmitted. Our recent molecular and proteomic studies revealed global differences in the expressed proteins of *E. chaffeensis* within different host cell environments [13]–[16]. The pathogen’s growth in different host cell environments is also a major contributor for its dual host adaptation and persistence [11]. The host cell-specific differences in the expressed proteins support the hypothesis that *E. chaffeensis* employs novel strategies to adapt and persist in both types of hosts, however, the exact mechanism of adaptation remains to be established.

In this study, we investigated ultrastructural differences in *E. chaffeensis* replicating in vertebrate and tick cells by employing transmission electron microscopy (TEM) analysis to assess if the organism differs in its progression. Specimens for TEM were prepared and observed under various magnifications ranging from 2,000× to 70,000 ×. The pathogen progression stages described here included the attachment of the organism to the host cell membrane, its engulfment, replication within a morula by binary fission, and release of the organisms from infected host cells by complete host cell lysis or by exocytosis. We found evidence for unique host cell-specific differences in the organism’s progression within phagosomes. In addition, our novel data suggest that *E. chaffeensis* enters into host nuclei.

## Results

### Morphological forms of *E. chaffeensis*

**Figure 1:**
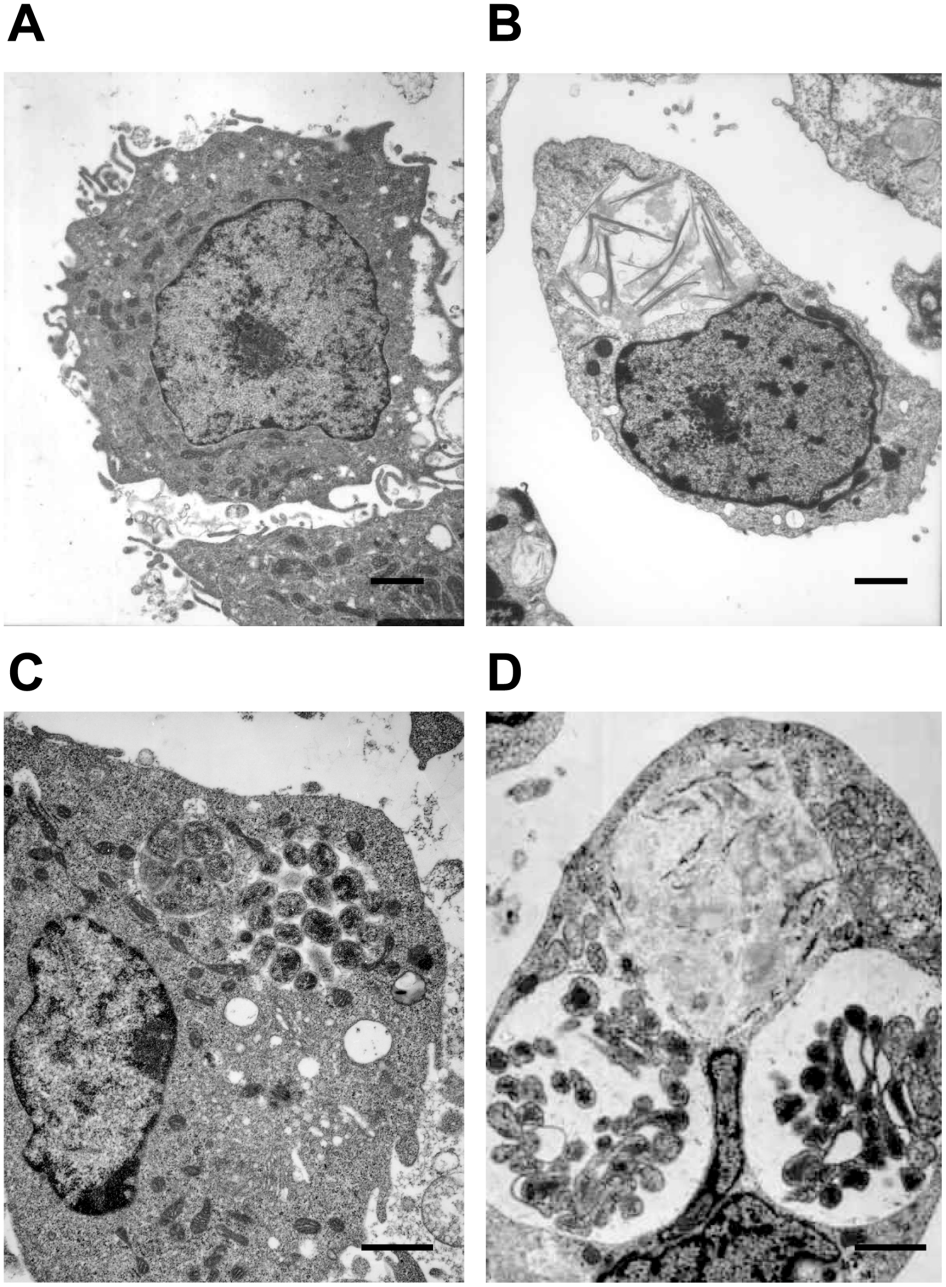
Two morphologically distinct cell forms of *E. chaffeensis* in infected macrophages or tick cells. This Figure included TEM images to represent uninfected macrophages (A) and tick cells (B) and *E. chaffeensis*-infected macrophages (C) and tick cell (D). Majority of the morulae in the infected macrophages harbored only reticulate cells or dense core cells. In infected tick cells, considerably more infected cells contained both cell forms of the bacteria within the same morula. (Scale bar 1 µm).

Transmission electron microscopic (TEM) analysis aided the visualization of diversity in the size and number of morulae containing *E. chaffeensis* organisms within invertebrate and vertebrate host cells. Two morphologically distinct forms (reticulate and dense core cells) were identified within the phagosomes of infected tick cells and macrophages. Although the two morphological forms observed for the first time for *E. chaffeens*is infection in tick cells, they are similar to the TEM data reported earlier for the organism in macrophage cultures [17]–[20]. The reticulate bodies had an even distribution of cytoplasmic structures, while the dense core cells contained condensed material considered to contain ribosomes and nucleoid material [17], [18]. *E. chaffeensis* in macrophages was relatively more synchronized compared to infected tick cells (Figure 1). About 38% of macrophage cells harbored only reticulate cells, 42% contained only dense core cells, and 20% of the cells observed contained both cell forms of *E. chaffeensis* but were found in separate morulae. The cells containing both forms in macrophage cultures were typically found in the later time points (96 and 168 hours), whereas the early time points (48–72 hours) contained primarily reticulate form. On the contrary, considerably a greater percent of the infected tick cells (34%) contained both cell forms of the bacterium within the same morula.

**Figure 2:**
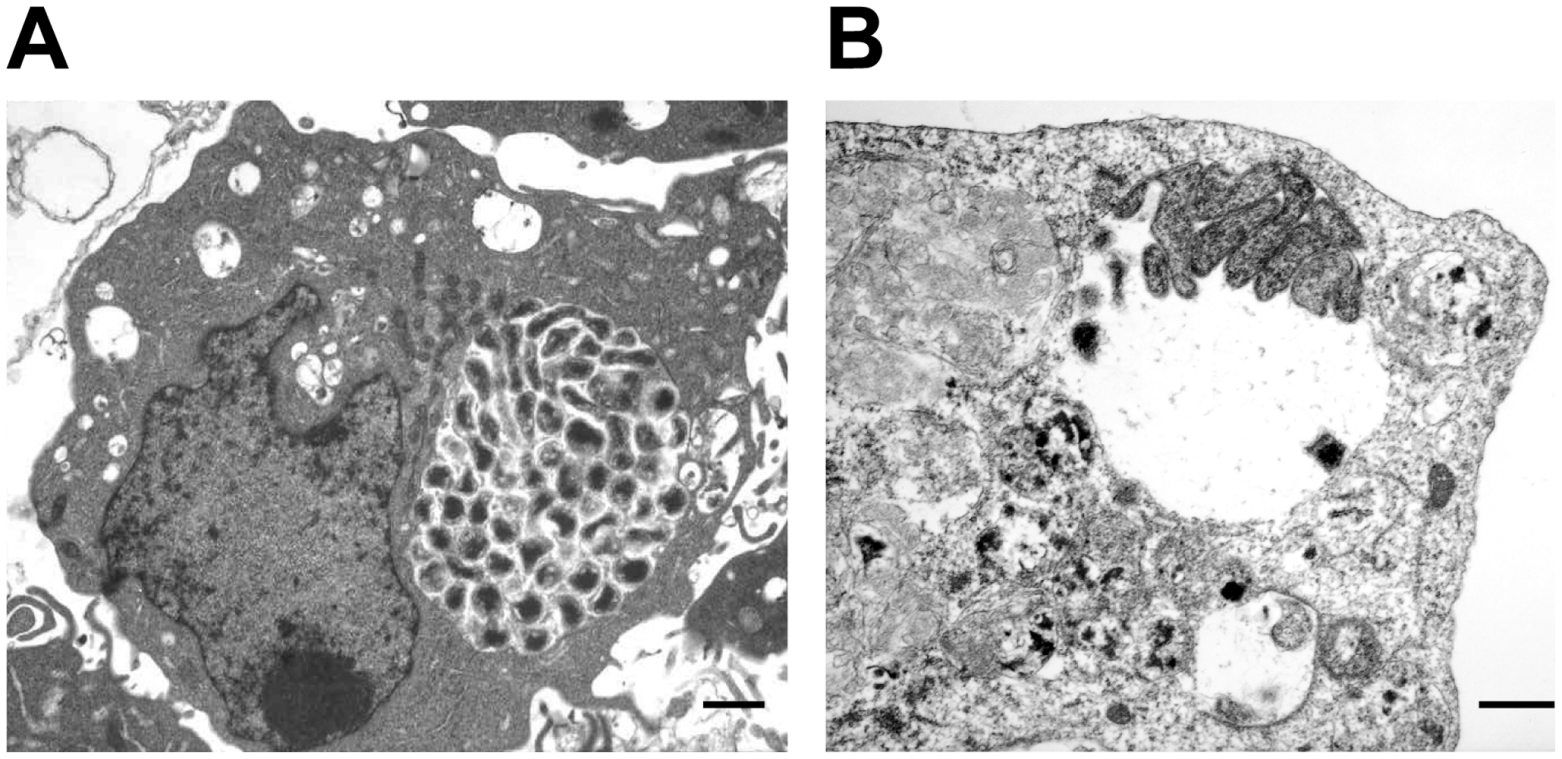
Variations of morulae in infected macrophages and tick cells. *E. chaffeensis* containing phagosomes within the infected macrophages (A) were more compact with organisms occupying most of the intra-morulae space. The organisms in infected tick cells (B) were mostly aggregated at one end of the morula or attached to the morula membrane, intra-morulae space is also considerable more in the tick cell phagosomes and the morula size is also larger. (Scale bar 1 µm).

**Figure 3:**
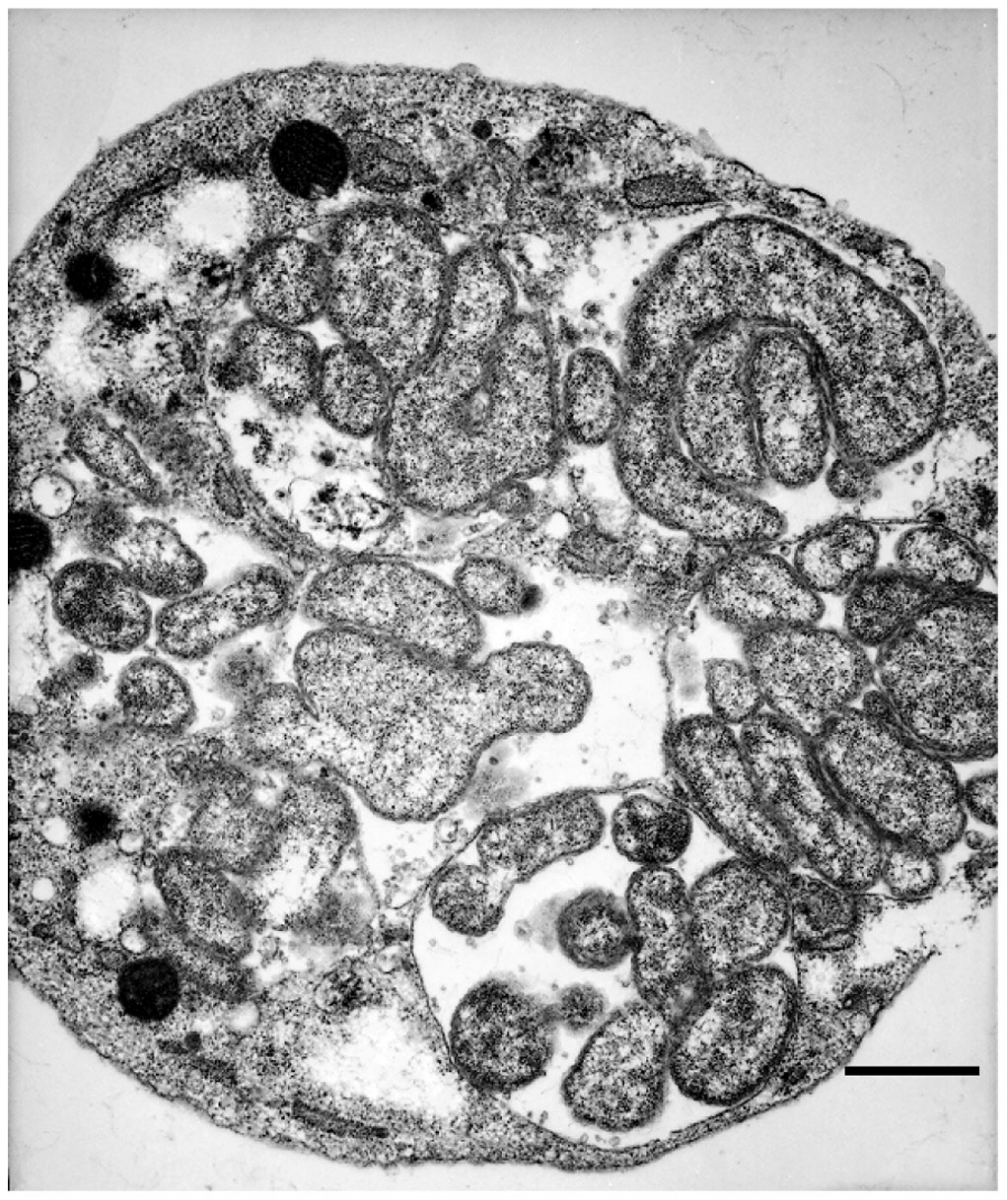
Extensive pleomorphic structures of *E. chaffeensis* in infected tick cells. *E. chaffeensis* in infected tick cells have extensive pleomorphic structures. (Scale bar 1 µm).

**Figure 4:**
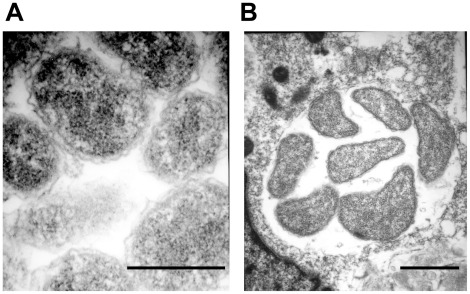
Corrugated outer membrane present in *E. chaffeensis*. Both *E. chaffeensis* reticulate and dense core forms have ruffled outer membrane structures (Higher magnification of reticulate forms from infected macrophages (A) and infected tick cells (B) are presented. (Scale bar 1 µm).

The intracellular vacuoles were filled with varying numbers of bacteria which appeared to range from one organism to greater than 100 organisms. In cells that contained large morulae, the host cell nucleus was characteristically pushed to one side. The characteristic morula membrane appeared as smooth, but the morula containing several bacteria had more ruffled membranes (Figure 2A). The morulae within the infected macrophages were more compact with organisms occupying most of the intra-morula space. In contrast, the organisms in infected tick cells were mostly loosely packed and dispersed throughout the phagosome (74%). In about 24% of the infected tick cells, the organisms aggregated at one end of the morula or attached to the morula membrane (Figure 2B). The morula size within the infected tick cells was also bigger, often occupying the majority of the cytoplasmic space (Figure 2B). Reticulate forms of *E. chaffeensis* within the tick cells were highly pleomorphic (Figure 3). The bacterium in both macrophages and tick cells contained two clearly visible membranes; the outer membrane and the inner membrane (Figure 4). The outer membrane was corrugated and was more prominent in the reticulate forms.

### Aggregation of Mitochondria Around a Morula

**Figure 5:**
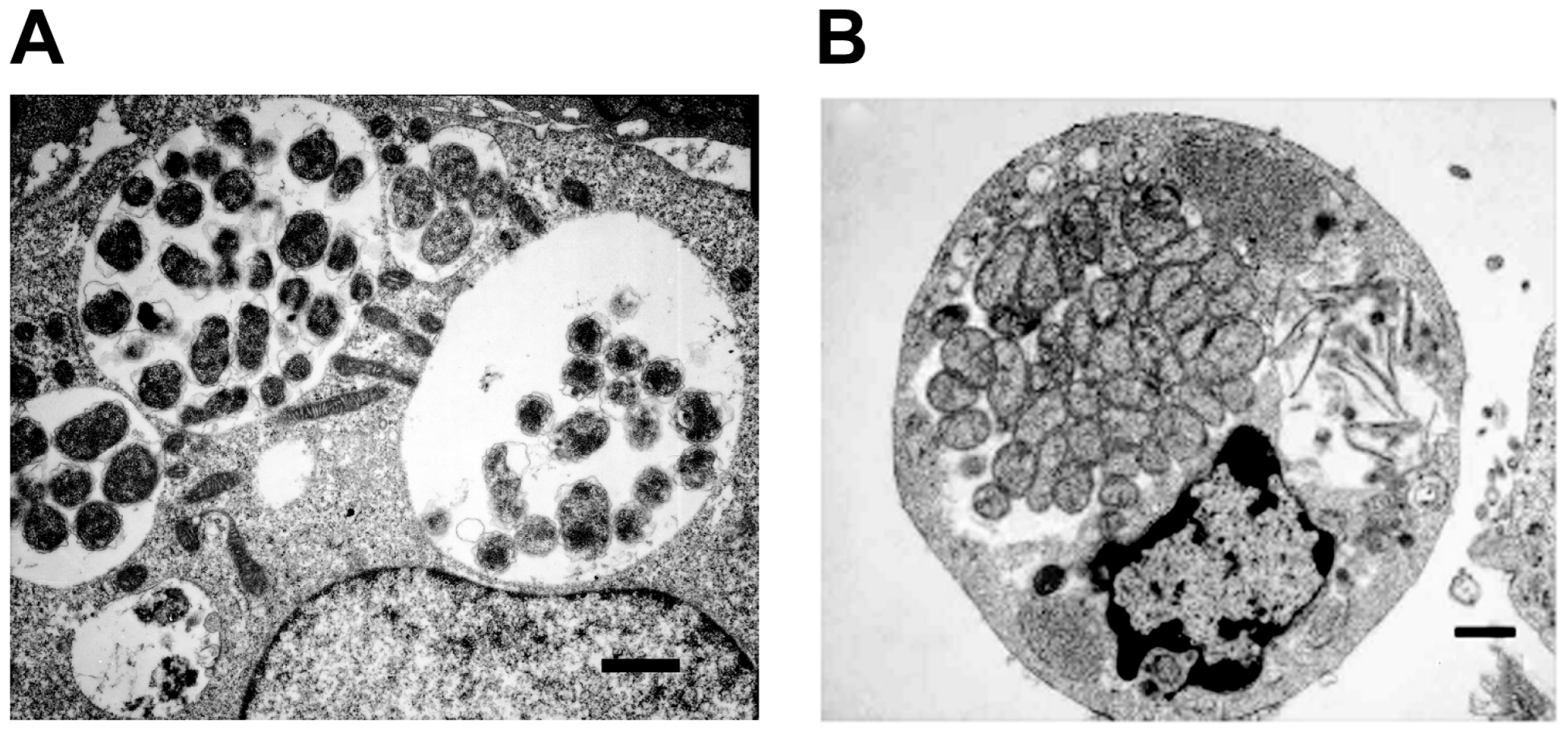
Mitochondria aggregation around morulae. Aggregation of mitochondria was observed more frequently in infected macrophages where they were also attached to the phogosomal membrane (A). Fewer mitochondria were visible in the infected tick cells harboring *E. chaffeensis* (B). (Scale bar 1 µm).

**Figure 6:**
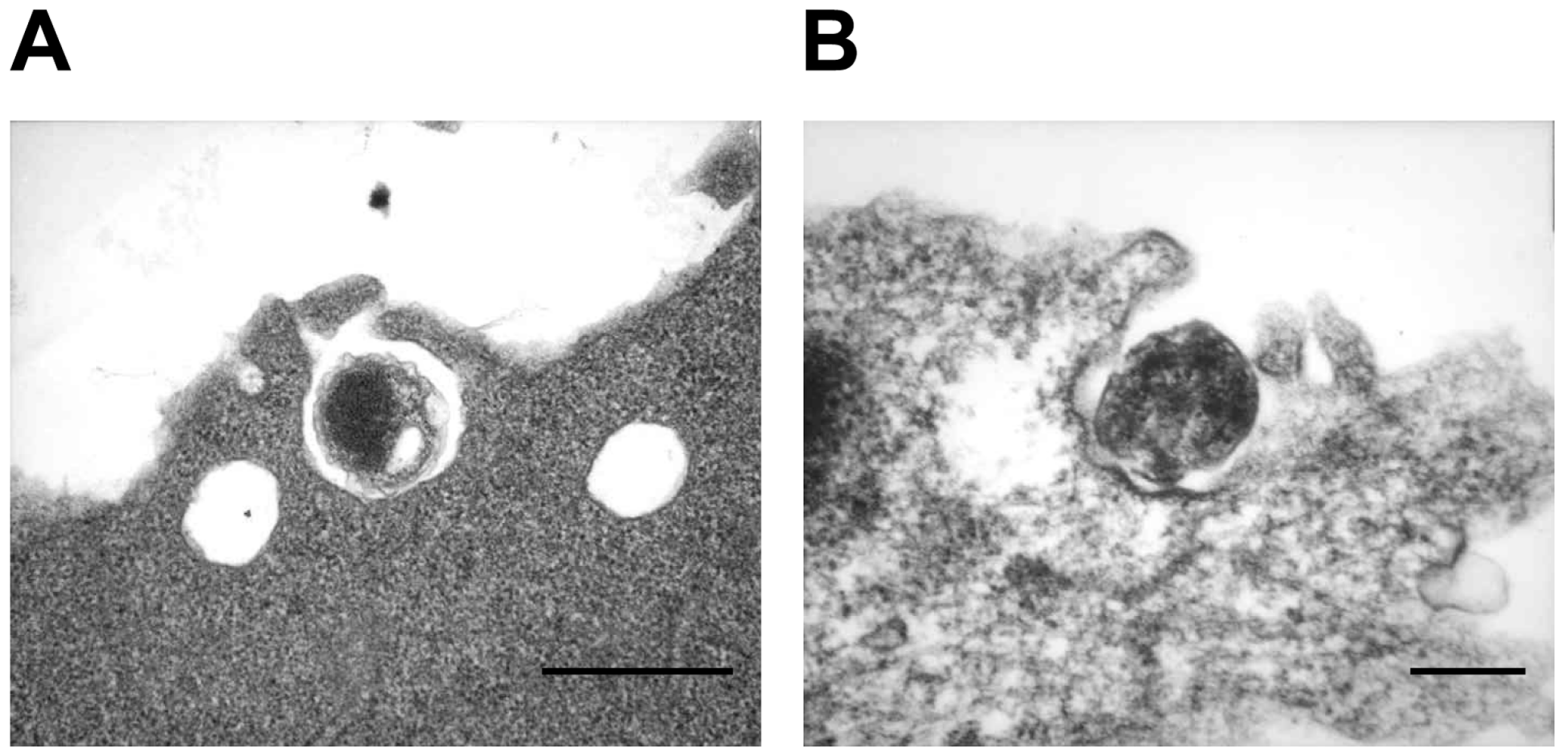
Attachment and internalization of dense core forms of *E. chaffeensis*. In macrophage cells (A), *E. chaffeensis* dense core cells interaction with the host cell membrane was seen as direct attachment to the host cell and with pseudopodia formed. *E. chaffeensis* dense core forms in tick cells (B) also attach to the host cell membrane and get internalized with the formation of pseudopodia. (Scale bar 1 µm).

Morulae containing *E. chaffeensis* organisms within the infected macrophage cells were often observed as surrounded with several mitochondria. In many infected macrophage cells, mitochondria were either in direct contact with a morula membrane or within the same vicinity of a morula (Figure 5A). In infected macrophages, 98% had mitochondria aggregated around a morula; nearly half of the cells having aggregated mitochondria also have in direct contact with a morula membrane, and only in 2% of the cells mitochondria could not be seen in the surrounding area of a morula. Contrary to this, fewer mitochondria were seen surrounding a phagosome containing *E. chaffeensis* organisms in tick cells (Figure 5B). TEM analysis revealed the attachment of *E. chaffeensis* dense core forms to the host cell membrane at earlier time points following infection. The organisms were also seen within the pseudopodia extensions from a host cell enabling engulfment and internalization into a phagosome (Figure 6A and B). The modes of attachment and engulfment appeared to be the same for the vertebrate and invertebrate cells.

### Cell Divisions and Release

**Figure 7:**
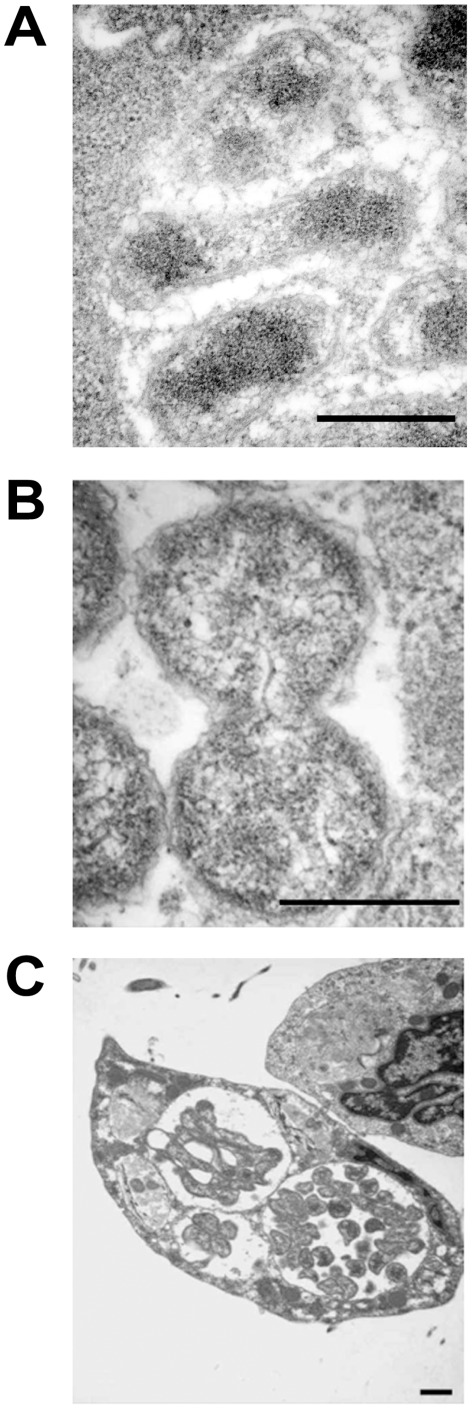
*E. chaffeensis* replication. *E. chaffeensis* reticulate cells in macrophages and tick cells exhibiting replication by binary fission (A, macrophage and B, tick cells). Tick cell grown organisms also included filamentous type cell divisions (about 20% of the cells) (C). This observation can also be seen in the image presented in Figure 1D. (Scale bar 1 µm).

**Figure 8:**
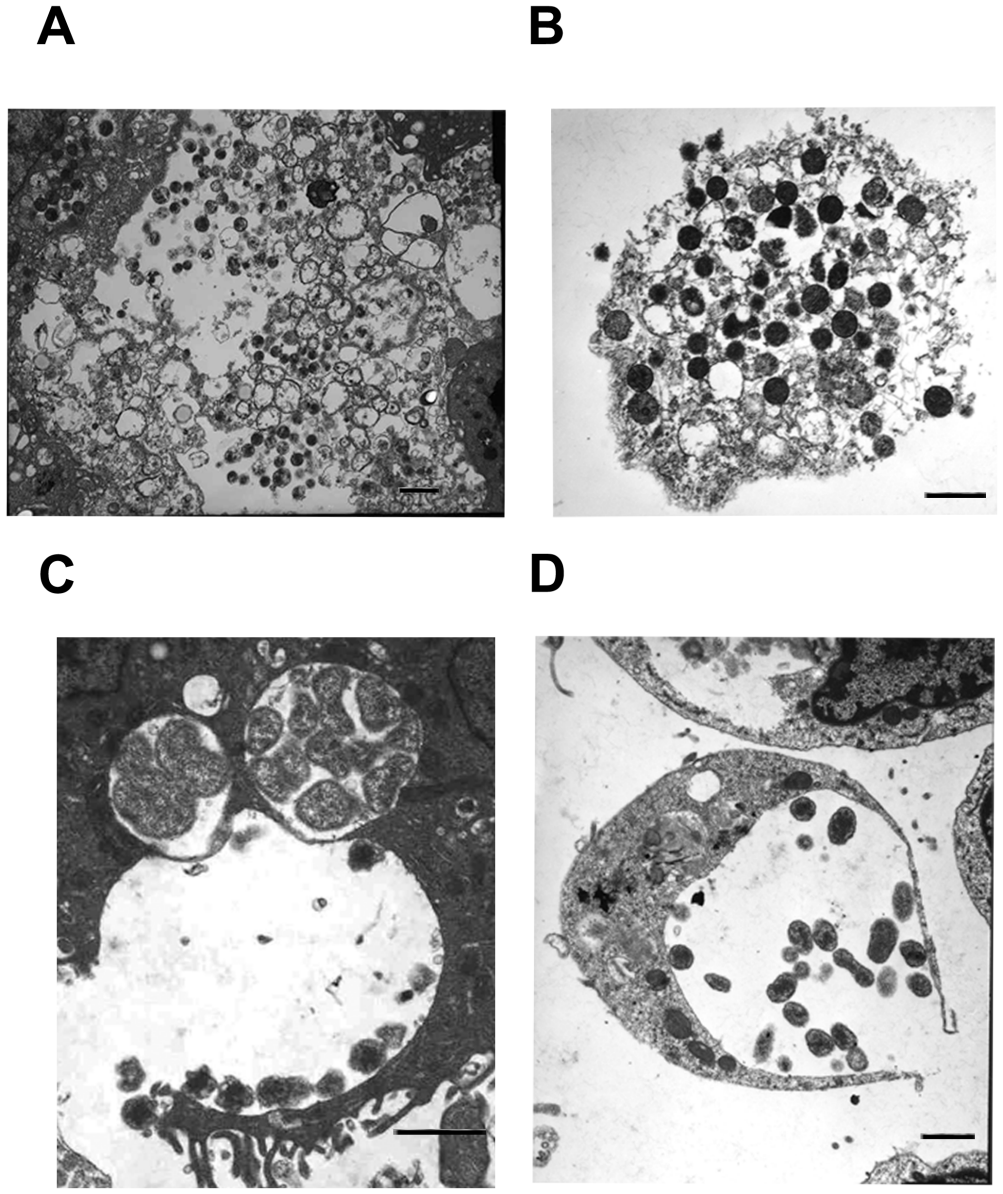
Release of *E. chaffeensis* from infected macrophages and tick cells. Most of the infected host cells exhibited release by complete lysis. A subset of the infected cells also released organisms by exocytosis by creating an opening to the morula membrane. (A and C, infected macrophages and B and D, infected tick cells) (Scale bar 1 µm).

**Figure 9:**
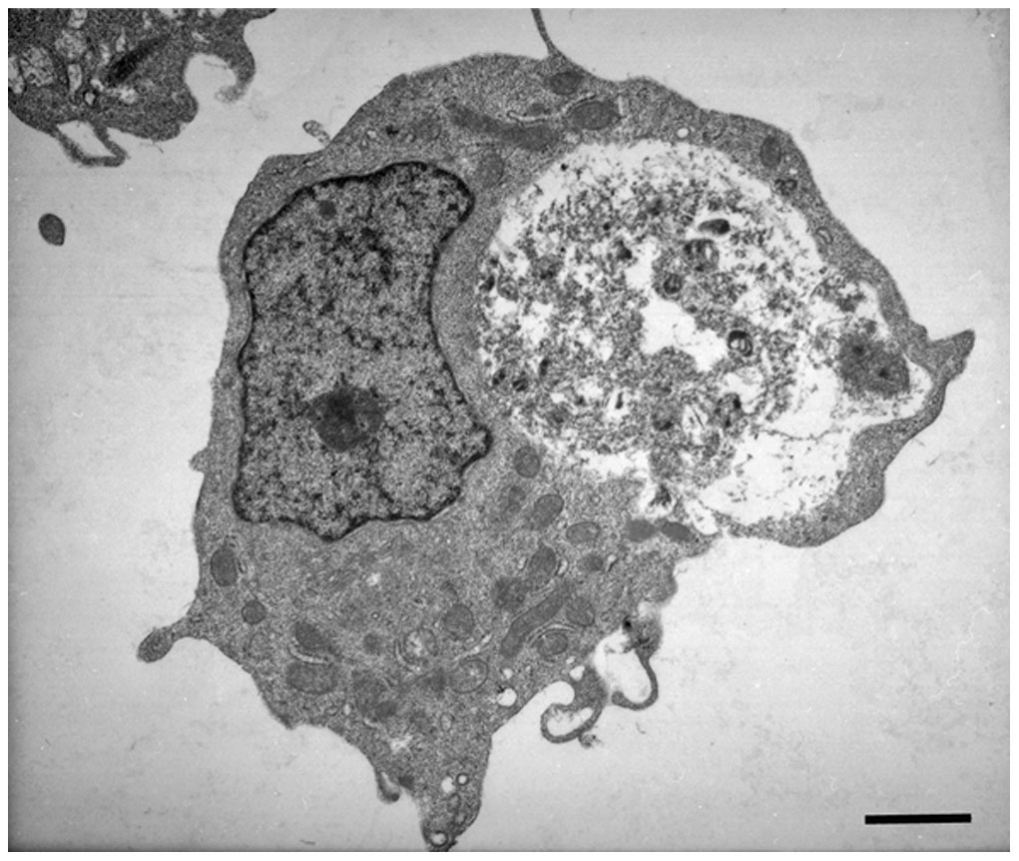
An infected macrophage containing phagosomes with cell debris. (Scale bar 1 µm).

The cell division process visualized in macrophages was typical of binary fission of reticulate cells (Figure 7A). This observation is consistent with the previous reports describing the TEM analysis of infected macrophages [17], [18], [20]. In tick cells, *E. chaffeensis* was also found to be dividing mostly by binary fission (80%) (Figure 7B). In addition, filamentous type cell divisions were observed in about 20% of infected cells (Figure 7C) (also can be seen in the image presented in Figure 1D). Once the bacteria have replicated to the point where the morula occupied majority of the space within a host cell cytoplasm, the organisms were released from the host cells mostly by complete lysis (90%) of the infected cells (Figure 8A and B). The release of bacteria in vertebrate and tick cells was also observed by exocytosis with an opening to a phagosomal membrane (Figure 8C and D). The organisms released by host cell lysis represented only dense core cells. About 5% of the infected macrophages, but not infected tick cells, also contained morulae that had organisms appeared to have been degraded (Figure 9).

### Invasion of Host Cell Nucleus

**Figure 10:**
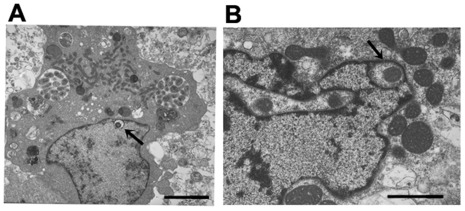
Inclusion in the nuclei of infected host cell. Vacuoles with inclusions within the infected macrophage (A) and tick cell (B) nuclei. (Scale bar 1 µm).

Bacteria of the family Anaplasmataceae reside within a phagosomal vacuole, whereas the related bacteria of the family Rickettsiaceae escape from a phagosome and reside in a host cell cytoplasm or move to the host cell nucleus [21]. To date, there were no reports that described any species within the family Anaplasmataceae to invade a host cell nucleus. Initial electron microscopic studies revealed the presence of vacuoles with inclusions in a subset of infected cell nuclei. As the infection progressed, the cells were more heavily infected and by 168 hours post infection about 18% of the infected cells (both macrophage and tick cells) also contained vacuoles within nuclei and included inclusions that resembled *Ehrlichia* organisms (Figure 10).

### Confocal Microscopy Analysis

**Figure 11:**
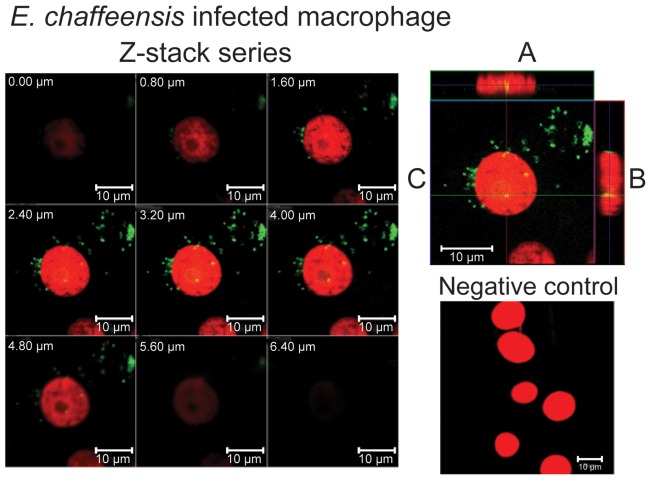
Confocal microscopy Z-stack imaging to localize *E. chaffeensis* within a host cell nucleus. Immunofluorescent detection of *E. chaffeensis* was accomplished with mAb 56.5. Detection was made using Alexa Fluor 488 (green fluorescence) anti-mouse secondary antibody. Propidium iodide was used to stain nuclei (red fluorescence). Yellow fluorescence indicates *E. chaffeensis* localized within the nucleus. (Z-stack images collected at 0.8 µm sections were presented in the top left panel of the figure. The cell sections in the figure were identified with the section depth at the top left on each image. The magnification in each cell section was presented at the bottom right of each image by placing a 10 µm scale bar.) In top right panel; A is Z-projection in the X–Z direction, B is Z-projection in the Y–Z direction and the blue lines in A and B indicate the Z-depth of the 3.20 µm optical slice in C. The green and red lines in C indicate the orthogonal planes of the X–Z and Y–Z projection, respectively. Uninfected cells which were subjected to similar immunofluorescence analysis were used to serve as a negative control for this experiment (bottom right panel).

To examine if the inclusions in the nuclei of infected cells are E. chaffeensis organisms, infected macrophage and tick cell cultures were subjected to double immunofluorescence labeling and confocal microscopic examination (Figure 11). Confocal microscopy offers the ability to optically slice through a cell and generate Z or depth information about a specifically labeled organism within a cell. By showing sequential optical slices through a cell (Figure 11) and generating orthogonal projections of all slices in a Z-series (A and B of the top right panel of the figure) it was possible to determine if *E. chaffeensis* localized in a nucleus. In both cell lines, nuclei were labeled with the nucleic acid stain propidium iodide (red in all images). Polyclonal sera made against the whole *Ehrlichia* organism [11] or three different monoclonal antibodies (mAbs) which recognize p28-Omp 19 protein (mAbs 56.5, 18.1, and 65.1) [22] were used as primary antibodies, followed by Alexa 488 (green color) conjugated secondary antibody to identify *E. chaffeensis* within the host cells (Figure 11 included data generated using mAb 56.5). Alexa 488 staining was visible in the inclusions of both cytoplasm (green) and nucleus (green-yellow) of infected macrophages. Similar analysis with infected tick cells exhibited obvious E. chaffeensis inclusions only in the cytoplasm, but not in the nucleus (not shown). The inclusions were observed in about 10% of the total cell population examined.

### Cell fractionation and Western Blot Analysis

**Figure 12:**
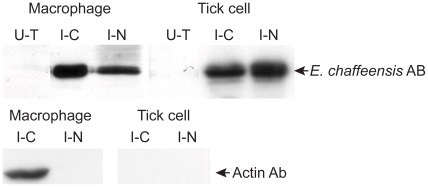
Western blot analysis to identify *E. chaffeensis* proteins. Total cell lysates from uninfected cells, cytoplasm (C) and nucleic (N) fractions from *E. chaffeensis*-infected macrophages and tick cells were assessed by immunoblot analysis using *E. chaffeensis* mAb 56.5 that recognizes p28 Omp 19 [22]. *E. chaffeensis* infected macrophage and tick cell protein fractions were also probed with β actin Ab. (U–T, uninfected cell-derived total soluble proteins; I–C, *E. chaffeensis*-infected cell derived cytoplasmic proteins; I–N, *E. chaffeensis*-infected cell derived nucleic proteins).

To further verify the presence of *E. chaffeensis* in the nucleus of infected macrophages and tick cells, infected cells were fractionated to cytosolic and nucleic fractions. Total proteins recovered from the nucleic and cytosolic fractions were isolated and subjected to Western blot analysis using polyclonal sera or using mAbs 56.5 that recognize p28 outer membrane protein [22] (Figure 12). *Ehrlichia* proteins were recognized in both the nucleic and cytosolic fractions derived from macrophages and tick cells, whereas the antibodies against the bacterium did not recognize similar proteins from the total cell extracts prepared from uninfected cells. To rule out the contamination of cytosolic proteins in the nucleic fraction, immune blot analysis was also performed for the nucleic and cytosolic fractionated proteins resolved from infected macrophages and tick cells using a mAb that recognizes canine β actin. The beta actin-specific antibodies identified a 42 kDa protein band only in the cytosolic fraction derived from infected macrophages.

## Discussion

TEM analysis of *E. chaffeensis* in macrophages and other vertebrate cell lines has been reported previously [17]–[20]. In particular, previous studies have been focused primarily on the organisms replicated in vertebrate cells, but there were no studies reported about how the organisms progress in infected tick cells. The current study represents the first detailed investigation describing the ultra-structures of *E. chaffeensis* in tick cells. This study also reevaluated and compared the organism’s morphological structures in vertebrate macrophages with those observed in infected tick cells. The study revealed several similarities in *E. chaffeensis* replication in macrophages and tick cells. In addition, the organism possessed several tick cell-specific differences. Here, we examined the invasion, replication within phagosomes, morphological variations in the replicating cells, and their subsequent release from the infected cells. Reticulate cells are typically larger and have an even distribution of the cytoplasmic material for *E. chaffeensis* cultivated in both macrophages and tick cells. The dense core cells contained a more compact and condensed material. Both cell forms were observed as having the characteristic corrugated membrane that has been described previously in other ultrastructure studies of the pathogens from the Anaplasmataceae family [17], [18], [23]–[27]. The dense core cells are the only form observed in the extracellular environment. This observation is consistent with a recent study demonstrating that the dense core cells represent infectious organisms [20]. Our analysis revealed for the first time that the reticulate forms of *E. chaffeensis* differ considerably in their morphology when replicating in two different host cell backgrounds. Specifically, the reticulate form in tick cells is often larger in size and is highly pleomorphic compared to those observed in macrophages.

The majority of *E. chaffeensis* organisms within a morula of vertebrate cells are either dense core or reticulate forms. The presence of both cell types in infected tick cells is relatively high compared to those observed in macrophage cells and may indicate that all *E. chaffeensis* organisms in a tick cell may mature at different times to form dense core cells, whereas in macrophages, the organisms grow more synchronously. In most of the morulae within infected tick cells, *E. chaffeensis* organisms are not tightly packed as observed in infected macrophages. Previous studies with *A. marginale*, *E. equi*, *E. canis*, *E. muris*, and the *A. phagocytophilum* have visualized vesicle like structures within the phagosomes where the organisms are replicating in vertebrate cells [18], [28], [29]. Likewise, Popov et al. [17] also reported similar vesicles within a phagosome of *E. chaffeensis* replicating in morulae of infected macrophages. Similar structures were also observed in the current study in the morulae of both infected tick cells macrophage cells (these structures can be seen in several images presented in this manuscript). It is not clear what these vesicles represent and their significance to the organism.

Our study identified mitochondria directly attached to a morula membrane or near the vicinity of a morula in infected macrophages. Similar observations are also reported for several other rickettsias in the Anaplasmataceae family [17], [18], [30], [31]. Together, these observations suggest that the organisms may require close interaction with a mitochondrion, possibly to obtain energy sources directly from the organelle. *E. chaffeensis* is an obligate intracellular pathogen and may depend heavily upon the host cell for energy. If this should be the case, a specific carrier mediated membrane transport system for exchange of ATP and ADP should be expressed at the surface of the morulae membrane to aid in the exchange of the nucleotides. ATP/ADP transporter proteins have been characterized for other intracellular bacteria, including *Rickettsia prowazekii, Chlamydia trachomatis*, and *Caedibacter caryophilus* [32]. *E. chaffeensis* genome includes several putative transporter proteins [33]. However, it remains to be studied if any of the transports are involved in support of the nucleotide uptake by the organism. In a recent study, Liu et al. [34] reported the selective inhibition of mitochondria function in *E. chaffeensis* infected vertebrate cells. The closer association of mitochondria, together with the inhibition of mitochondria function reported earlier, suggests that the interactions between the morulae and mitochondria may be necessary for the pathogen’s survival in the phagosomal environment.

Our TEM analysis of *E. chaffeensis* infected macrophages and tick cells revealed distinct developmental stages. We visualized *E. chaffeensis* to have active interaction with host cell projections in both macrophages and tick cells. Phagocytosis is the likely mechanism by which *E. chaffeensis* enters into both vertebrate and invertebrate host cells. The TEM examination revealed adhering of organisms to host cell membrane and support the hypothesis for receptor mediated endocytosis. This hypothesis is consistent with the reports suggesting that the 120 kDa outer membrane protein of *E. chaffeensis* expressed predominantly on the surface of the dense core cells and aides in the attachment to host cells [19], [20].

Previous reports suggest that the dense core cells of *E. chaffeensis* transform to reticulate cells prior to the organisms replication by binary fission within the phagosomes of infected macrophages [6], [17], [18]. In the current study, we observed bacterium forms having filamentous structures with short spherical organisms attached to the structures. These observations suggest that the bacterium also replicates by filamentous type cell divisions that is similar to *Mycoplasma* species [35]. Previous studies reported the absence of lipopolysaccharide (LPS) on *E. chaffeensis* [36], which is further supported by the absence of genes encoding proteins needed for the LPS in the bacterial genome [33]. The pleomorphic nature of reticulate cells and filamentous structures, observed in the current study in infected tick cells, may also have resulted due to the lack of this cell wall substance.

Release of organisms from infected cells was observed as a result of the complete lysis of the host cells or by exocytosis of the organisms from morulae releasing the bacteria from an intact host cell. The release of bacteria by exocytosis observed for *Ehrlichia* is similar to other intra-phagosomal pathogens such as *Chlamydia* species [37]–[39]. The dense core cells are the only cell form observed in the released *E. chaffeensis* organisms that were also attached to naïve cells. Together, these data support the prior observations that the dense core cells are the only infectious forms [20]. Overall, the developmental cycle visualized in both macrophage and tick cells included the attachment, replication by binary fission and/or by filamentous type cell divisions (tick cells), and finally the release of dense core bacteria by total cell lysis or by exocytosis for subsequent infection to naïve host cells. Recently, Thomas et al. [37] presented evidence that the *E. chaffeensis* are transported to neighboring cells through the host cell filopodium during initial stages of infection, a form of exocytosis. This appears to be one of the mechanisms by which the bacterium infects naïve cells. We, however, did not find similar host cell filopodium containing *E. chaffeensis* organisms in our TEM studies.

In this study, we also found evidence of two novel observations: 1) a subset of infected macrophages appear to clear *E. chaffeensis* from phagosomes, and 2) the presence of vacuoles in the host cell nucleus with *E. chaffeensis* organisms. We identified a subset of cultured macrophages that contained phagosomes with cellular debris, but did not include *Ehrlichia* organisms. The cellular debris may represent degraded bacterial organisms. This observation suggests that a subset of macrophages is capable of clearing *E. chaffeensis* from their phagosomes. Considerable evidence is presented in the literature that *E. chaffeensis* infected animals, including humans, do induce the strong B and T cell responses [13], [40]–[42]. If the infection was not processed by vertebrate macrophages or by other antigen presenting cells, one cannot expect a host response in inducing acquired immune response. It is possible that a subset of antigen presenting cells *in vivo* also breakdown *E. chaffeensis* organisms and that the immunogenic epitopes are presented for the induction a cellular response.

The second novel observation in this study was the identification of inclusions in the nuclei of a subset of *E. chaffeensis* infected host cell. We presented three lines of evidence demonstrating the entry of *E. chaffeensis* organisms into host cell nucleus; TEM, confocal microscopy and Western blot analysis. It is not clear how the bacterium enters into the host cell nucleus. One possible mechanism could be that the organisms may be trapped in a host cell nucleus during the cell divisions. Alternatively, the organisms may actively gain entry by nuclear phagocytosis. These hypotheses remain to be verified. It is also not clear if the bacterium indeed enters into the nucleus of an infected host cell under *in vivo* conditions. Inclusions in the vacuoles within the nucleus of a subset of infected cells are similar in size to *E. chaffeensis* organisms. Confocal microscopic analysis and Western blot analysis further confirmed the TEM observations that the inclusions observed within the nucleus of a subset of infected cells were indeed *E. chaffeensis* organisms. In our Western blot analysis, we presented clear evidence that the nuclear extracts are not contaminated with cytoplasmic proteins for infected macrophages by demonstrating the presence of β-actin only in the cytoplasmic extracts. Similar experiment, however, could not provide conclusive evidence of tick cell infection, as the β-actin antibody used in the current study does not cross-react with tick protein homologs. Although the nuclear protein extraction method was the same for fractionating proteins from infected macrophages and tick cells, the possibility of contamination of tick cell nuclear extract with *Ehrlichia* proteins from cytoplasmic extracts cannot be excluded. Thus, the bacterial localization with the nucleus of tick cells remains to be validated further.

The inclusions found in the nuclei are approximately one micron in size suggesting that the organisms found in the nuclei of infected cells do not appear to be replicating. This is the first study to document the presence of *E. chaffeensis* organisms in nuclei of infected cells of both vertebrate macrophages and tick cells. Recent studies suggest that the *Ehrlichia* and *Anaplasma* species pathogens transport bacterial proteins, such as the AnkA repeat proteins, into the infected host cell nuclei [43]–[45]. The proteins appear to bind to nuclear DNA and alter host gene expression. It is not clear if our observation that the inclusions in a host cell nucleus has any biological significance.

In summary, we reported ultrastructure variations of *E. chaffeensis* in vertebrate macrophages and in infected tick cells. Both infected vertebrate and tick cells contained two morphological cell forms (reticulate and dense core forms), the dense core form attached to the host cell membrane to gain entry by phagocytosis, transform to reticulate form and replicates within a morula by binary fission, convert to dense core form and release into the extracellular environment as a result of whole cell lysis or by exocytosis. We identified *E. chaffeensis* cultivated in tick cells to contain larger reticulate forms and have a higher degree of pleomorphism. They also included filamentous like structures, possibly resulting from replications similar to *Mycoplasma* species [35]. We have presented two novel findings; cell debris in phagosomes of a subset of infected macrophages, which possibly represent degradation of the organisms, and the localization of *E. chaffeensis* organisms within the nucleus of a subset of infected host cells. The morphological differences in infected tick cells and macrophages parallel to our prior observations that *E. chaffeensis* organisms express unique host cell specific proteins [13]–[16]. The morphological differences in the pathogen’s progression in infected macrophages and tick cells are further evidence that the pathogen employs unique host-cell specific strategies.

## Materials and Methods

### Cultivation of *E. chaffeensis*

*E. chaffeensis* (Arkansas isolate) was propagated in the canine macrophage cell line (DH82) using the minimal essential medium (MEM) supplemented with 6.5% fetal bovine serum and 2 mM L-glutamine at 37°C with 5% CO_2_ essentially as described earlier [46]. DH 82 is a macrophage-monocyte cell line from a dog with malignant histiocytosis [38] and is commonly used for *in vitro* cultivation of *E. chaffeensis* [14], [46]. The ISE6 tick cell line, an embryonic cell line of *Ixodes scapularis* described previously [25], was also used to cultivate *E. chaffeensis* as we reported earlier [14]. Briefly, uninfected and infected tick cell cultures were maintained at 34°C in L15B300 medium modified with 5% tryptose phosphate broth, 5% heat-inactivated fetal bovine serum, and 0.1% bovine lipoprotein concentrate at pH 7.2. The medium for infected cultures was additionally supplemented with 25 mM HEPES and 0.25% NaHCO_3_ with an adjusted pH of 7.5 [25]. The intracellular growth of the organisms was monitored with a polychromatic staining kit, Hema-3 stain (Fisher Diagnostics, Middletown, VA) following the transfer of 100 µl of culture suspension onto a slide by cytospin centrifugation (Wescor Inc., Logan, UT).

### Preparation of *E. chaffeensis* Cultures for Use in Electron and Confocal Microscopy Analysis

*E. chaffeensis* infected culture at about 80–90% infectivity were harvested from a confluent T75 flask and centrifuged at 2000×g for 10 min to remove intact host cells and nuclei. The culture supernatant was filtered through 5 and 3 µm filters (Millipore, Billerica, MA) to recover host cell-free *E. chaffeensis* organisms. The filtered solution was centrifuged at 15,500×g for 15 minutes and the host cell free bacteria were resuspended in 5 ml each of minimal essential medium (for vertebrate culture) or L15B medium (tick cell culture). One milliliter each of the culture was used to inoculate naïve 5 ml DH82 or ISE6 cultures. Infected cultures were harvested at different time points post infection. Cultures were harvested by centrifuging at 500×g for 5 min at 4°C and the pellets were resuspended in 1× phosphate buffered saline (PBS) for use in electron microscopy analysis.

### Transmission Electron Microscopy Analysis

All centrifugation steps used in preparing the TEM samples were performed at 4°C for 5 min at 200×g, unless otherwise specified. The cultures in PBS were fixed with 1 ml of Karnovsky’s fixative containing 2% paraformaldehyde, 2.5% gluteraldehyde in 0.1 M cacodylate buffer (pH7.4) at 4°C overnight. The cells were then washed three times with 1 ml of 0.1 M cacodylate buffer and were incubated in 1 ml of 1% osmium tetraoxide in 0.1 M cacodylate buffer for one hour at 4°C, washed thrice with double distilled water and then resuspended in 2% trypsin soy agar solution. Each sample was diced with a teflon coated razor blade and placed in a wheaton glass vials with 50% ethanol at room temperature for 15 min, then stained with 70% ethanol/uranyl acetate in the dark for one hour at room temperature. Cells were passed through a dehydration process with an ethanol gradient of increased concentrations from 50% to 100%. All samples embedded in the resin were transferred to silicon molds to allow for polymerization to be completed. All blocks were examined under a dissecting scope to identify a sample that was flush to the end of the block using an Ultracut E-Reichert-Jung ultramicrotome, sections of 0.5 µm were cut in the range of 75–90 nm, and placed on Athene Thin Bar copper grids (Ted Pella, Redding, CA). The grids were stained with uranyl actetate in 70% ethanol followed by Reynold’s lead citrate. The grids were stained with lead citrate and the stained grids were examined under a Hitachi H-300 electron microscope (Hitachi High-Tech, San Jose, CA). Images were captured on Kodak Electron Microscopy film 4489 (Electron Microscopy Sciences, Hatfield, PA) and developed in Kodak D-19 Developer (Electron Microscopy Sciences, Hatfield, PA) as per the manufacturer’s instructions. The photograph scale marker was used to identify magnification.

### Quantitative Analyses of TEM Images

Typically, individual cells viewed under TEM were counted to determine various observations. Twenty separate grids were captured from each micrograph to access differences for infection in tick cells and macrophages.

### Immunolabeling

*Ehrlichia* infected macrophage or tick cell cultures (140 µl) were transferred to a glass slide (Fisher Scientific, Pittsburg, PA) by using a cytospin centrifuge (Wescor Inc., Logan, UT). The slides were air dried and fixed in 4% paraformaldehyde for 20 min at room temperature. The slides were then washed with PBS. Antibodies were diluted in FA serum diluting buffer (VMRD, Inc., Pullman, WA) and 10 µl of either polyclonal antisera (1∶256) raised in mice against *E. chaffeensis* [11] or one of the three different monoclonal antibodies (mAbs) (1∶500) that recognize 28 kDa outer membrane protein, p28-Omp 19, (mAbs 18.1, 56.5 or 65.1) [22] were transferred to the slides. Slides were then placed in a moist chamber at 37°C for 30 min, washed in 1× FA buffer and placed in a jar containing the same solution for 10 min. Ten microliters of Alexa Flour 488 conjugated goat anti-mouse IgG (H+L) (0.5 ng/ml) (Invitrogen, Carlsbad, CA) was added to each slide and placed in a moist chamber at 37°C for 30 min and were rinsed as described above. Cells were then permeabolized by adding 10 µl of 0.1% Triton X-100 in 1X PBS. For visualization of the nuclei, 10 µl of 1.5 µM propidium iodide (PI) in PBS was added to the slide. The slides were air-dried and mounted with Fluoromount-G (Fisher Scientific, St. Louis, MO). The samples were then stored at 4°C in the dark until viewing.

### Confocal Microscopy

Samples were viewed on a Zeiss laser scanning confocal microscope model LSM 5 PASCAL equipped with an Axioplan 2 MOT Research Microscope using a 63×/1.4 oil Plan apochromat objective. Single track images of Alexa 488 and PI labeling as well as multi-track images, track #1 Alexa 488 and PI images and track #2 reflected light images, were collected. For single track images and multi-track images, Alexa 488 and PI fluorophores were excited with the 488 nm line of a 25 mW Argon laser and the 543 nm line of a 1 mW HeNe laser, respectively, and fluorescence imaged using an HFT 488/543/633 primary dichronic, an NFT 545 secondary dichronic, a 560 long pass filter and channel 1 (photomultiplier tube, red) for viewing PI, a 505–530 band pass filter and channel 2 (photomultiplier tube, green) for imaging Alexa 488. The pinhole for channel 1 was set to an Airy unit of one and channel 2 was adjusted to an optical slice thickness equal to channel 1. Single and multi-track Z-stacks were collected at 0.8 µm intervals through the full thickness of infected cells to determine if *E. chaffeensis* resided within the nucleus.

### Protein Subcellular Fractionation and Western Blotting Analysis

*E. chaffeensis* protein fractions were prepared from infected macrophage and tick cell cultures having 80–90% infectivity. The protein fractions from the whole cell, cytosol, and nuclei were isolated using ProteoExtract™, a Subcellular Proteome Extraction Kit, (Calbiochem, Darmstardt, Germany) according to the manufacturer’s instructions. The extracted protein fractions were resolved on 12% SDS-PAGE gel, transferred on to a nitrocellulose membrane for use in Western blot analysis. Western blot analysis was performed with *E. chaffeensis* mAb 56.5 [22] or polyclonal sera obtained from infected C57BL/6J mice as described earlier [11]. Immuno blot analysis was also performed using polyclonal antibodies against mouse beta actin which cross react with canine β-actin (catalog# AB6276, Abcam, Cambridge, MA).
